# Merging microarray studies to identify a common gene expression signature to several structural heart diseases

**DOI:** 10.1186/s13040-020-00217-8

**Published:** 2020-07-08

**Authors:** Olga Fajarda, Sara Duarte-Pereira, Raquel M. Silva, José Luís Oliveira

**Affiliations:** 1 IEETA/DETI, University of Aveiro, Aveiro, 3810-193 Portugal; 2 Department of Medical Sciences and iBiMED-Institute of Biomedicine, University of Aveiro, Aveiro, 3810-193 Portugal; 3 Current Address: Universidade Católica Portuguesa, Faculdade de Medicina Dentária, CIIS-Centro de Investigação Interdisciplinar em Saúde, Campus de Viseu, Viseu, 3504-505 Portugal

**Keywords:** Heart disease, Random forest, Gene expression signature, Microarray data

## Abstract

**Background:**

Heart disease is the leading cause of death worldwide. Knowing a gene expression signature in heart disease can lead to the development of more efficient diagnosis and treatments that may prevent premature deaths. A large amount of microarray data is available in public repositories and can be used to identify differentially expressed genes. However, most of the microarray datasets are composed of a reduced number of samples and to obtain more reliable results, several datasets have to be merged, which is a challenging task. The identification of differentially expressed genes is commonly done using statistical methods. Nonetheless, these methods are based on the definition of an arbitrary threshold to select the differentially expressed genes and there is no consensus on the values that should be used.

**Results:**

Nine publicly available microarray datasets from studies of different heart diseases were merged to form a dataset composed of 689 samples and 8354 features. Subsequently, the adjusted *p*-value and fold change were determined and by combining a set of adjusted *p*-values cutoffs with a list of different fold change thresholds, 12 sets of differentially expressed genes were obtained. To select the set of differentially expressed genes that has the best accuracy in classifying samples from patients with heart diseases and samples from patients with no heart condition, the random forest algorithm was used. A set of 62 differentially expressed genes having a classification accuracy of approximately 95% was identified.

**Conclusions:**

We identified a gene expression signature common to different cardiac diseases and supported our findings by showing their involvement in the pathophysiology of the heart. The approach used in this study is suitable for the identification of gene expression signatures, and can be extended to different diseases.

## Background

Heart disease is the leading cause of death worldwide [1] and in particular in the United States [2] and Europe [3]. The 2017 Global Burden of Disease estimated that ischaemic heart disease alone is responsible for approximately 8.93 million deaths globally, which represents an increase of 22.3*%* compared to 2007. Hypertensive heart disease, in turn, is estimated to be responsible for approximately 0.93 million deaths and has increased by 46.6*%* compared to 2007 [4]. A thorough understanding of heart disease can lead to the development of more efficient diagnosis and treatments that may prevent premature deaths.

A gene expression signature (GES) is a set of genes whose altered expression can distinguish patients with different conditions, e.g. healthy vs. diseased [5, 6]. GES can be used for diagnosis, prognosis or prediction of therapeutic response [7] and it can also assist drug discovery by helping to identify a new potential target [6]. Several studies identified GESs for specific heart conditions. Barth et al. [8] identified 27 genes that can distinguish patients with dilated cardiomyopathy from patients with nonfailing hearts. Kittleson et al. [9] compared the gene expression of hearts from patients with nonischemic and ischemic cardiomyopathy with those from patients with nonfailing hearts. They identified 257 genes differentially expressed in nonischemic cardiomyopathy and 72 genes in ischemic cardiomyopathy. Tan et al. [10] reported 103 genes that were differentially expressed between failing and nonfailing hearts in patients with end-stage dilated cardiomyopathy.

Microarray technology is widely used to measure, in a single experiment, the expression levels of thousands of genes simultaneously [11]. The development of next-generation sequencing led to the conception of a new technology to measure gene expression, the RNA-Sequencing (RNA-seq) [12]. Despite the advantages of RNA-seq, microarray technology continues to be widely used, due to its lower cost and the existence of mature, reliable and robust processes and analysis tools [13, 14]. Furthermore, as the scientific community recommends that the data generated should be publicly available [13], several repositories were created. The Gene Expression Omnibus (GEO) [15] at the National Center for Biotechnology Information (NCBI) and the ArrayExpress [16] at the European Bioinformatics Institute (EMBL-EBI) nowadays provide a tremendous amount of microarray data available for further analysis.

Most microarray datasets are composed of a limited number of samples, and therefore, have low statistical power to identify a GES [17]. A way to obtain more reliable results is by merging microarray datasets from independent studies, since this leads to an increase of sample size [18]. However, merging microarray datasets is challenging, since most of the datasets were originated using different platforms measuring the expression of diverse sets of genes [19]. Furthermore, combining microarray datasets from different experiments introduces a batch effect to the data. Batch effect is the term used to identify technical, non-biological, variations introduced in the measurements due to the use of different processes, protocols and platforms [20]. This technical variation can obscure and confound true biological variation, leading to erroneous results [21].

GESs are commonly identified using statistical methods. Fold change and statistical tests like the *t* test are frequently used methods [22, 23]. The R/Bioconductor [24] software package limma [25] is also widely used and is considered one of the best methods to identify differentially expressed genes in comparison studies [26, 27]. This package implements linear models and empirical Bayes methods for microarray data analysis [28]. All these methods are based on the definition of an arbitrary threshold to select the GES and there is no consensus as to the values that should be used.

More recently, supervised machine learning algorithms have been applied to identify differentially expressed genes [29–32]. These algorithms have the leverage to construct a prediction model that can be applied to classify new samples. However, in a microarray experiment the number of features (the genes) is substantially higher than the number of samples and supervised machine learning algorithms can be inefficient when applied to high-dimensional datasets [33]. One way around the high-dimensional problem is reducing the number of features before applying supervised machine learning algorithms. Random forest is a supervised learning algorithm developed by Breiman [34] that constructs various decision trees, using for each split a random subset of features, and makes a prediction by combining the predictions of the different decision trees. It is dependent on only two tuning parameters, provides measures of variable importance and can be used directly for high-dimensional problems without reducing the number of features [35]. An empirical comparison of ten supervised learning algorithms performed by Caruana and Niculescu-Mizil [36] concluded that random forest was one of the algorithms that gave the best average performance.

The objective of this study was to identify a common GES in heart disease. To achieve this goal, we first merged nine publicly available microarray datasets from studies of different heart diseases. Then, we randomly divided the merged dataset into a training set and a test set and repeated this procedure 30 times, obtaining 30 training sets and 30 test sets. Subsequently, we used the R/Bioconductor software package limma to determine the adjusted *p*-value and the fold change for every training set. A set of adjusted *p*-value cutoffs combined with a list of different fold change thresholds were used to obtain several differentially expressed gene sets. To obtain a GES for every combination of adjusted *p*-value and fold change cutoff, we intersected the 30 sets of differentially expressed genes obtained using the 30 training sets. Afterwards, we evaluated the performance of every GES, on the 30 test sets, using the random forest algorithm and identified the one which had the best accuracy in classifying samples from patients with heart diseases and samples from patients with no heart condition. We identified a set of 62 differentially expressed genes with a classification accuracy of approximately 95%.

## Methods

The methodology used to obtain a GES for heart disease is described in this section, as well as the functional analysis performed.

### Data selection

All the datasets used are publicly available and were downloaded from GEO. The query: *((heart) OR cardio) AND (((disease) OR pathology) OR failure)* and the following filter criteria were used:

- Species: *Homo sapiens*;
- Sample types: heart tissue;
- Number of samples: more than 23 diseased or control samples (i.e. samples collected from heart donors with no previous history of heart disease);
- Access to unprocessed data (.cel files).

Nine gene expression datasets, with the following accession numbers, were selected: GSE1145 [37], GSE1869 [9], GSE2240 [38], GSE17800 [39], GSE21610 [40], GSE22253 [41], GSE42955 [42], GSE57338 [43] and GSE115574 [44]. A summary of the datasets is presented in Table 1, where, for each dataset, the platform, the number of samples and the heart diseases of the diseased samples can be found.

**Table 1:**
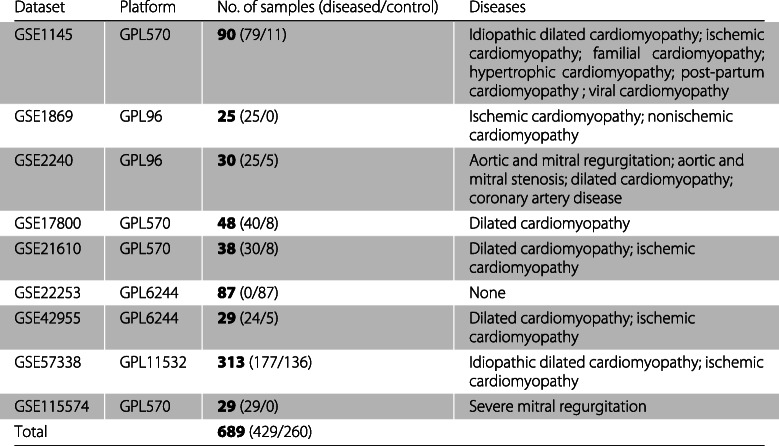
Summary of the nine datasets used in this study

Some original datasets had more samples than those used in this study and the reasons for excluding some samples are given below.

Data of the original dataset GSE1145 were collected using the Affymetrix Human Genome U133 Plus 2.0 array (GPL96) and the Affymetrix Human Genome U95 Version 2 array (GPL8300). The seventeen samples of platform GPL8300 were not used in this study because the gene list of this platform is substantially different from the gene list of the remaining platforms used. Regarding the dataset GSE2240, we did not use the five samples of patients which had diabetes mellitus, because this may alter the gene expression patterns. We also exclude the 30 samples of the original dataset GSE21610 which were collected after the implementation of a ventricular assist device (VAD), since the use of a VAD can alter the gene expression patterns.

Concerning the dataset GSE22253, we did not use the 21 samples which have rs1333049 genotype CC, because Pilbrow et. al [41] conclude that the risk allele associated with coronary heart disease is C. Finally, the original dataset GSE115574 is composed of 29 samples obtained from the left atrial tissue and 30 obtained from the right atrial tissue. Since the samples obtained from the left and right atrial tissue came from the same patients and most of the samples from the other datasets were obtained from the left ventricular tissue, this study only used the samples obtained from the left atrial tissue.

### Data pre-processing

Before merging the microarray datasets, the raw data (.cel files) must go through pre-processing. The raw data of the same platform were merged and we used the oligo package [45], of the R/Bioconductor software package, which implements the robust multichip average (RMA) pre-processing method [46], to perform background correction, normalization and probe summarization.

The microarrays used in this study are oligonucleotide microarrays and each probe corresponds to one or a set of short oligonucleotide sequences. In such arrays a gene can be represented by multiple sequences, i.e. multiple probes, and the expression measurements of these probes which represent the same gene may be very different [13]. We decided to remove these conflicting expression measurements, but in order not to significantly reduce the number of genes used in the study, we used, as probe identifier, the GenBank sequence accession identifier, which uniquely identifies a biological sequence. In Affymetrix Human Genome U133A Array (GPL96) and Affymetrix Human Genome U133 Plus 2.0 Array (GPL570), a probe is associated with a unique GenBank identifier, but in Affymetrix Human Gene 1.0 ST Array (GPL6244) and Affymetrix Human Gene 1.1 ST Array (GPL11532) a probe is associated with a list of GenBank identifiers. So, firstly, for each dataset obtained using platforms GPL96 and GPL570, all the probes corresponding to multiple or no GenBank identifier were removed. Concerning the dataset obtained using platforms GPL6244 and GPL11532, only the lists containing unique GenBank identifiers were maintained, all other lists being removed. Besides, we assigned to each GenBank identifier of a list the corresponding expression measurements of that list.

At this stage, we identified the common GenBank identifier across the different platforms and merged the datasets using only these common GenBank identifiers. The resulting merged dataset has 689 samples and 8354 features (the common GenBank identifiers).

### Feature selection

As observed previously, GESs are commonly identified using statistical methods, but these methods depend on the definition of an arbitrary threshold to select the GES. The cutoffs for the adjusted *p*-value commonly used are 0.01 and 0.05 [47]. Concerning the fold change, the cutoffs normally used are 1.5 and 2 [48] and between 2 and 3 [13, 23]. In this study, the merged dataset was randomly divided into a training set (70% of the samples) and a test set (the remaining 30%). This procedure was repeated 30 times and this way 30 different training sets and 30 different test sets were obtained. Next, the R/Bioconductor software package limma was used to determine in each training set, for each feature, the adjusted *p*-value (adjusted using Benjamini and Hochberg’s method to control the false discovery rate [49]) and the fold change. In this study, we used the adjusted *p*-value and the fold change combined and instead of a threshold, we used a list of thresholds to identify features which represent differentially expressed genes. Thus for the adjusted *p*-value, we used as thresholds the values 0.01 and 0.05 and for fold change, we used the values within the range 1.5–3, which correspond to log2 fold changes in the range of 0.585 and 1.585, approximately. In this way we obtained, for each training set, several sets of features that represent differentially expressed genes. For every combination of adjusted *p*-value and fold change cutoff, we intersected the 30 sets of features obtained using the 30 training set and get a feature set for every combination of adjusted *p*-value and fold change cutoff.

### Batch effect removal

The gene expression measurements may vary according to biological factors as well as non-biological ones, i.e. technical sources of variation, such as the use of different platforms or different processing times [50]. These non-biological variations are also called the batch effect. Several approaches exist to deal with the batch effect. Nygaard et. al [51] suggested that, when possible, the batch variable should be included in the statistical analysis. Therefore, when using the limma package we included the platform type as a covariate. Another approach is to adjust the data for batch effects before using the dataset and that is what we have done before using the random forest algorithm. We used the ComBat method [52] implemented in the sva package [53] to batch-adjust the gene expression data of the merged dataset.

### Random forest

The next step after batch-adjustment is to select from the various sets of features the one with the best predictive accuracy. Accuracy is the fraction of correct predictions and is determined as $\text {Accuracy} = \frac {\text {Number of correct predictions}}{\text {Number of prediction made}}$. We used the random forest algorithm to evaluate the predictive accuracy of the various sets of features and implemented it using the R package caret [54]. *Caret’s* random forest implementation has two parameters that can be fine-tuned, namely the number of trees in the forest (*ntree*) and the number of features in the random subset used in each split (*mtry*). To select the best parameters for every set of features, we used the training sets and repeated 10-fold cross-validation. The models were fine-tuned to maximize the accuracy. Combinations of the following values for each parameter were used:

- *ntree*: 125, 250, 375, 500, 625, 750, 875, 1000;
- *mtry*: 2, 3, …, *n*, where *n* represents the total number of features in a set.

The evaluation of the performance of the tuned models was done using the test sets. Besides accuracy, other metrics can be used to evaluate the model’s performance, namely balanced accuracy, specificity, precision, recall or sensitivity, the F1 Score, the Matthews correlation coefficient (MCC), the area under the ROC (receiver operating characteristic) curve (AUC) and the area under the precision-recall curve (AUCPR). These measurements are determined as:

$$\text{Balanced Accuracy} = \frac{1}{2} \times \left(\frac{\text{TP}}{\text{TP + FN}} + \frac{\text{TN}}{\text{TN + FP}}\right) $$

$$\text{Specificity} = \frac{\text{TN}}{\text{TN + FP}} $$

$$\text{Precision} = \frac{\text{TP}}{\text{TP + FP}} $$

$$\text{Recall} = \frac{\text{TP}}{\text{TP + FN}} $$

$$\text{F1 Score} = \frac{2 \times \text{Precision} \times \text{Recall}}{\text{Precision} + \text{Recall}} $$

$$\text{MCC} = \frac{\text{TP} \times \text{TN} - \text{FP} \times \text{FN}}{\sqrt{\text{(TP + FP)} \text{(TP +FN)} \text{(TN +FP)} \text{(TN +FN)}}} $$

where TP represents the number of diseased samples correctly classified; TN represents the number of control samples correctly classified; FP represents the number of control samples wrongly classified as diseased samples; and FN represents the number of diseased samples wrongly classified as control samples.

The ROC curve plots the recall as a function of 1-specificity at all classification thresholds and the AUC is the area under the ROC curve [55].

The precision-recall (PR) curve plots the recall as a function of precision at all classification thresholds. PR curves are more accurate in presenting the performance of models than ROC curves when the datasets used are unbalanced. The AUCPR is also known as the average precision [56]. Both AUC and AUCPR are summary metrics of the respective curves.

### Functional analysis

To investigate the functional meaning of the genes obtained with our approach, we performed a gene ontology (GO) enrichment analysis on the genes of the selected feature sets. We analyzed the up-regulated and down-regulated genes separately, using a statistical over-representation test (Fisher’s exact, False Discovery Rate correction) in the PANTHER Classification System 1 [57]. Next, we obtained the networks of protein-protein interactions (PPIs) of the up-regulated and down-regulated genes from the gene set with the best overall results. We used the STRING database 2 [58], and searched for data from text-mining, experiments, databases and co-expression, with a default median confidence level. Finally, we retrieved information from the DisGeNet database 3 [59] to assess which genes had previously been associated with disease, specifically cardiac-related diseases. From the file with all gene disease associations (GDA), we filtered “diseaseSemanticType” by “Disease or Syndrome” and then “diseaseName” by all containing “cardio*” or “cardiac” or “heart”.

## Results

To identify a GES common in heart disease by merging microarray studies, we used a methodology composed of several steps. The first step consisted of identifying the studies to merge. We identified 9 datasets, whose data were obtained using four different microarray platforms. Using different platforms involves pre-processing of the datasets before they can be merged. We used the GenBank accession identifier to identify the probes and exclude the probes corresponding to multiple or no GenBank identifier. Table 2 presents the number of GenBank identifiers remaining in each dataset, according to the corresponding platform.

**Table 2:** Number of GenBank identifiers remaining after the removal of repeated GenBank identifiers

Platform	GPL96	GPL570	GPL6244	GPL11532
No. of GenBank identifier	20079	48100	121142	57714

In platforms GPL6244 and GPL11532, the number of unique GenBank identifiers is higher than in the other platforms because in these two platforms the probes are associated with a list of GenBank identifiers.

**Fig. 1:**
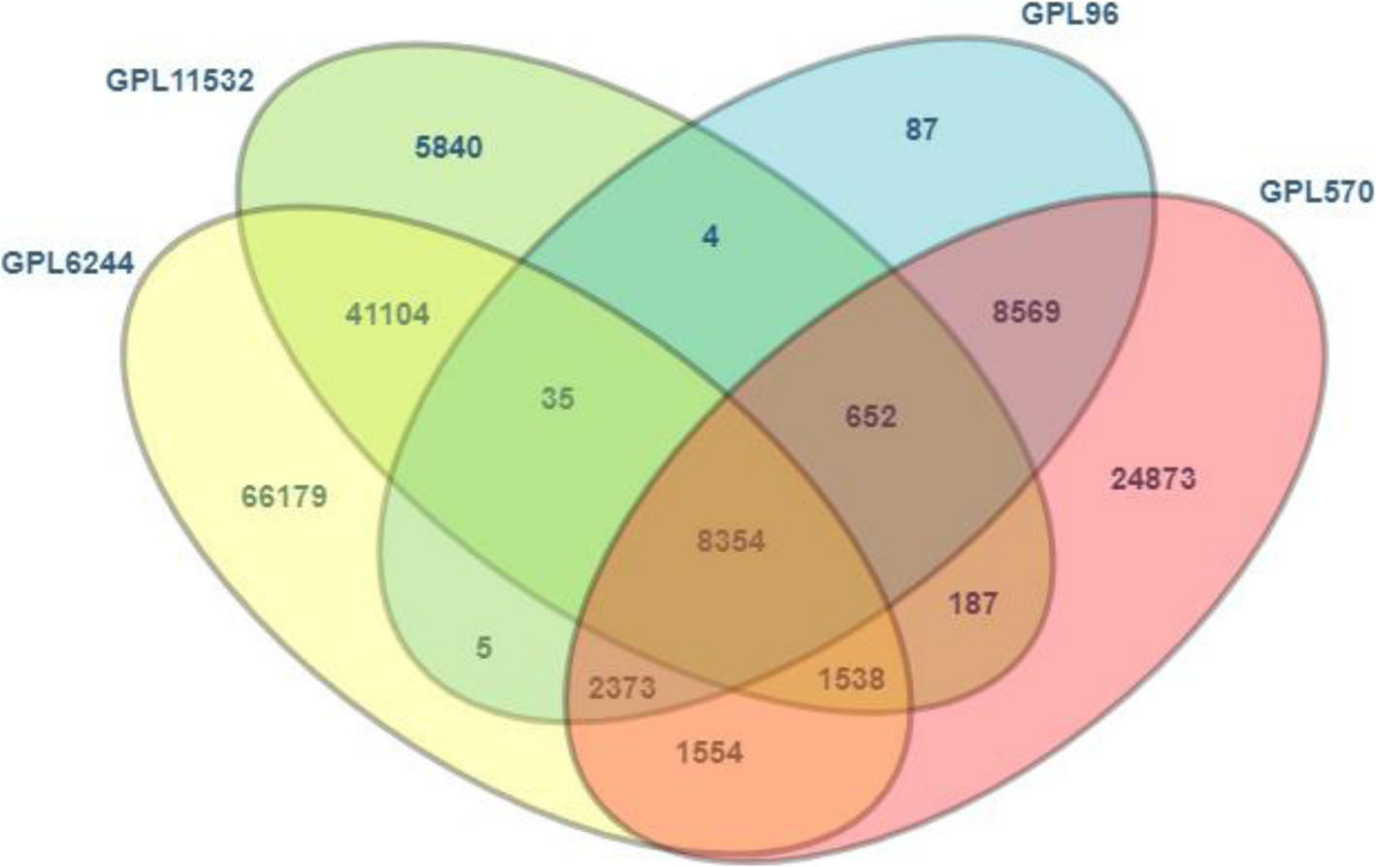
Venn diagram of common GenBank identifiers. The Venn diagram presents the overlap of GenBank identifiers across the four microarray platforms: GPL96, GPL570, GPL6244 and GPL11532

Before merging the datasets we determined the common GenBank identifier across the four platforms. Figure 1 presents a Venn diagram of the common GenBank identifier across the four platforms. The four platforms have 8354 GenBank identifiers in common and all the probes not corresponding to these GenBank identifiers were removed from the nine datasets.

As can be observed in Fig. 1, platforms GPL6244 and GPL11532 are more similar to each other than to the other two, having 51031 GenBank identifiers in common. Platforms GPL96 and GPL570 are also more similar to each other, having 19948 GenBank identifiers in common.

After merging the various datasets to form a common dataset with 689 samples and 8354 features, we randomly divided the merged dataset into a training set and a test set and repeated this procedure 30 times, obtaining 30 different training and 30 different test sets. Then, we used the R/Bioconductor software package limma to obtain several sets of features for every training set. To get a unique feature set for every combination of adjusted *p*-value and fold change cutoff, we intersect the 30 features sets obtained using the 30 training sets. We observed that for a fold change within the range 1.5–3, we obtained the same sets of features using a cutoff for the adjusted *p*-value of 0.01 and of 0.05. Additionally, for fold changes within the range 2.7–3 the features sets had a very small number of genes (less than five) and therefore we choose not to use these feature sets.

For each fold change threshold used and a *p*-value of 0.01 we obtained a set of different features, resulting in a total of 12 different sets. As the fold change threshold increases, the number of features decreases. So for a fold change cutoff of 1.5 we obtained a set of 95 features and for a fold change cutoff of 2.6 we obtained a set of 7 features.

**Fig. 2:**
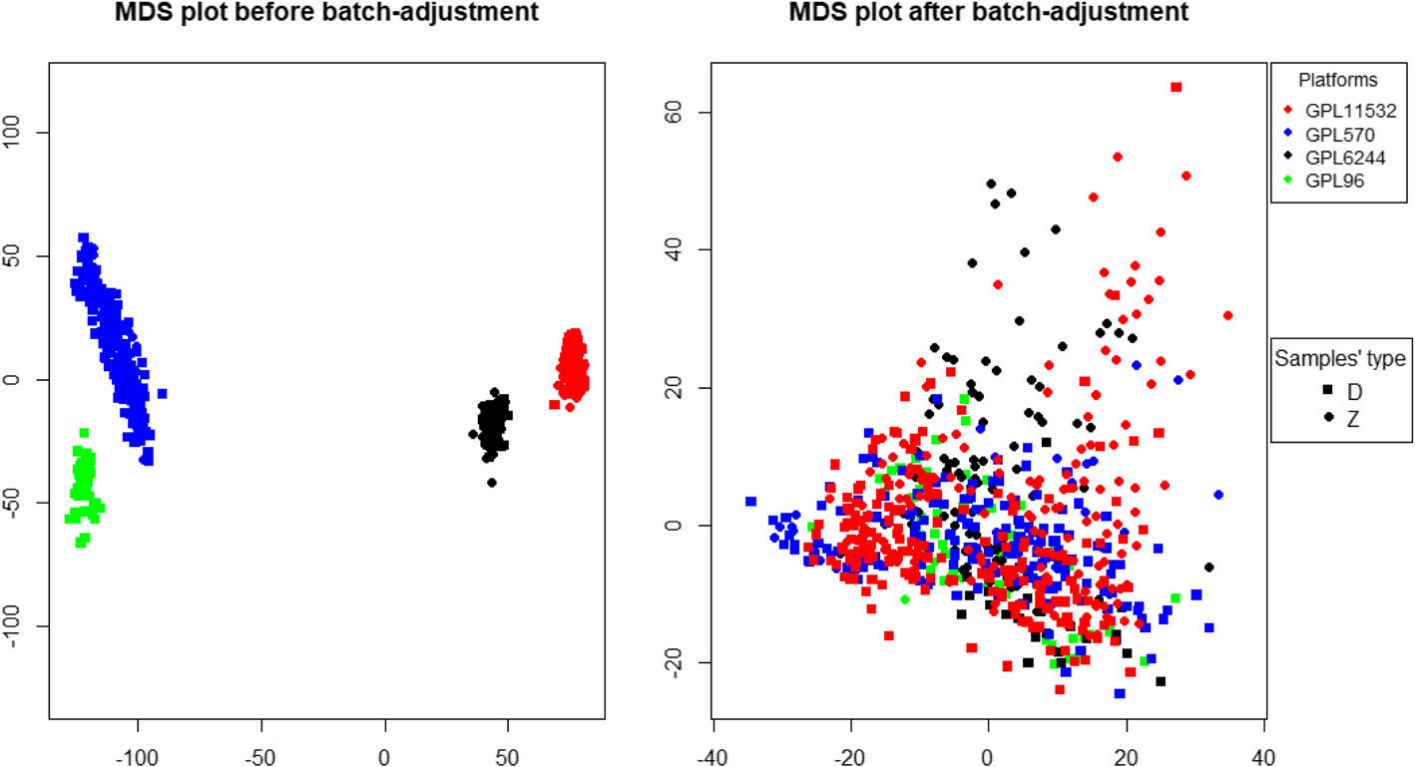
MDS plot before and after batch-adjustment. MDS was performed using the 689 samples of the merged dataset. Before batch-adjustment four clusters of samples driven by the four platforms can be observed. After batch-adjustment no cluster can be observed

To evaluate the predictive accuracy of each set of features we used the random forest algorithm. However, before applying the random forest algorithm, a batch-adjustment to the data was required. Figure 2 presents the multidimensional scaling (MDS) plot showing the distribution of the merged dataset before and after the batch-adjustment. As can be observed before the batch-adjustment there are four clusters corresponding to the four platforms which disappear after the batch-adjustment.

The parameters *ntree* and *mtry* of the random forest were fine-tuned using a set of different values and using the training sets. To evaluate the performance of the tuned models we used the test sets.

Table 3 presents for each set of features, the number of features, the correspondent number of genes, the mean and the 95% confidence interval (95%-CI) of the accuracy and balanced accuracy of the model when applied to the test sets.

**Table 3:**
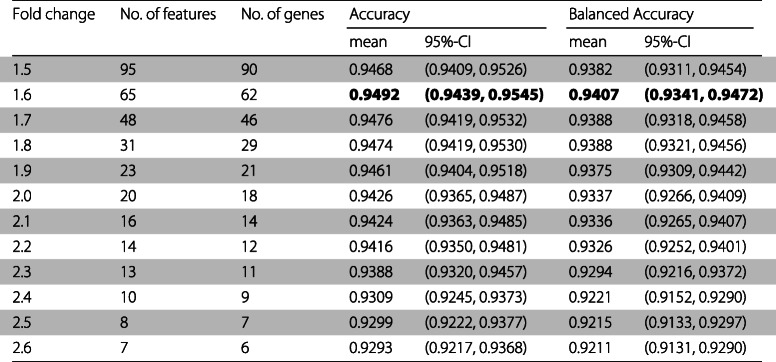
For every fold change, the number of features, the number of genes, the accuracy and the balanced accuracy of the classifier

Table 4 presents for every set of features the mean and the 95% confidence interval of the specificity, precision, and recall for the model when applied to the test sets. Table 5 presents the mean and the 95% confidence interval of the F1 score, the MCC, and the mean and the 95% confidence interval of the AUC and the AUCPR are presented in Table 6.

**Table 4:**
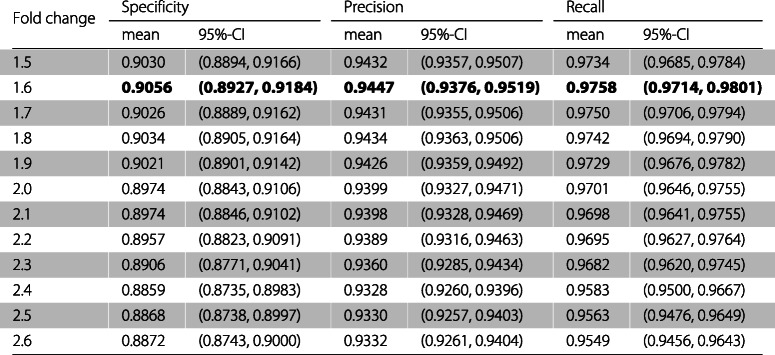
For every fold change the mean and the 95% confidence interval of the specificity, precision, and recall of the classifier

**Table 5:**
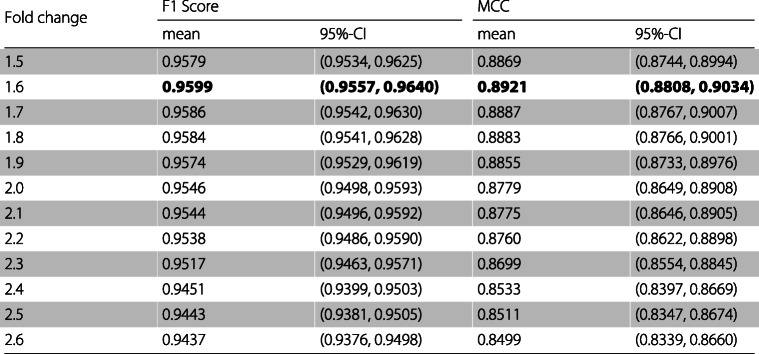
For every fold change the mean and the 95% confidence interval of the F1 score and MCC of the classifier

**Table 6:**
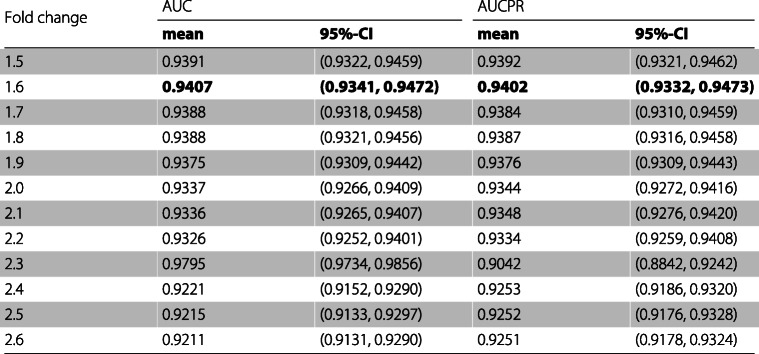
For every fold change the mean and the 95% confidence interval of the AUC and the AUCPR of the classifier

As can be observed in Tables 3, 4, 5, and 6 the feature set obtaining the best mean accuracy (approximately 95%), mean specificity, mean precision, mean F1 score, mean MCC, mean AUC and mean AUCPR is the one using a fold change cutoff of 1.6.

The feature set using a fold change cutoff of 1.6 is composed of 65 GenBank identifiers which correspond to 62 genes. 37 are up-regulated (fold change >1.6) in heart disease and 25 of these genes are down-regulated (fold change $<\frac {1}{1.6}\simeq 0.625$).

Table 7 presents the 37 up-regulated genes from the feature set obtained using a fold change cutoff of 1.6, as well as the respective mean adjusted *p*-value, the mean fold change and the mean variable importance obtained. Table 8 presents the 25 down-regulated genes along with the respective mean adjusted *p*-value, the mean fold change and the mean variable importance obtained. The mean adjusted *p*-values and the mean fold changes presented in Tables 7 and 8 were obtained by averaging the values obtained using the 30 training sets. The variable importances are determined by measuring the mean decrease accuracy using the out-of-bag samples. Variables with a larger importance measurement are more important for classification [60]. The mean variable importances presented in Tables 7 and 8 were obtained by averaging the values obtained using the 30 test sets.

**Table 7:**
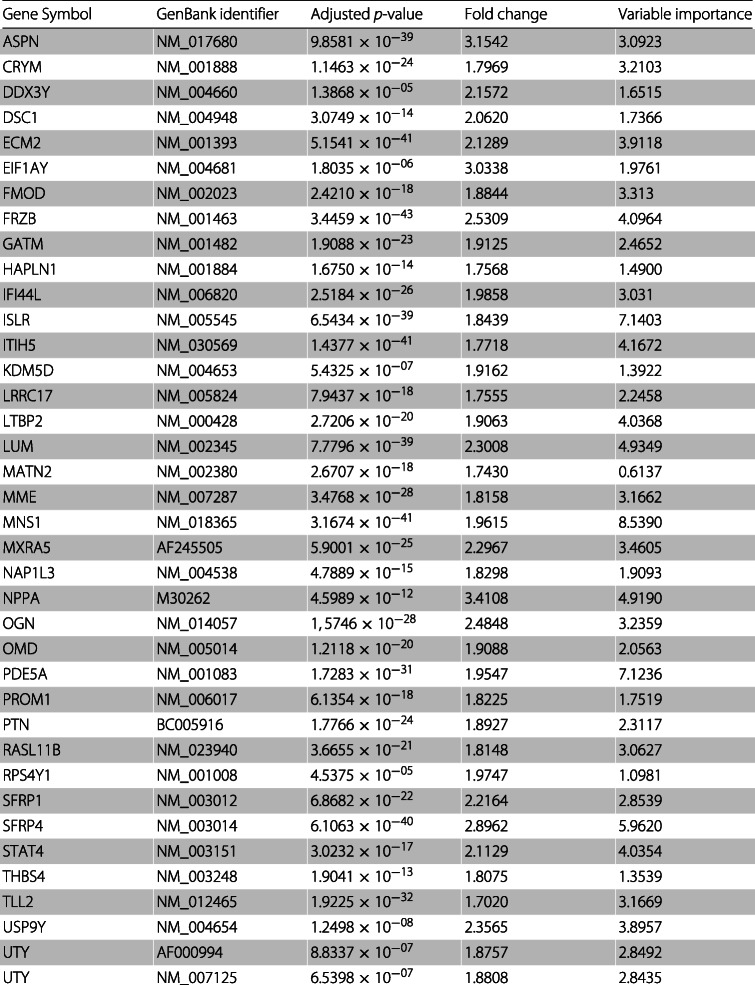
Common up-regulated genes in heart disease obtained when using a fold change cutoff of 1.6

**Table 8:**
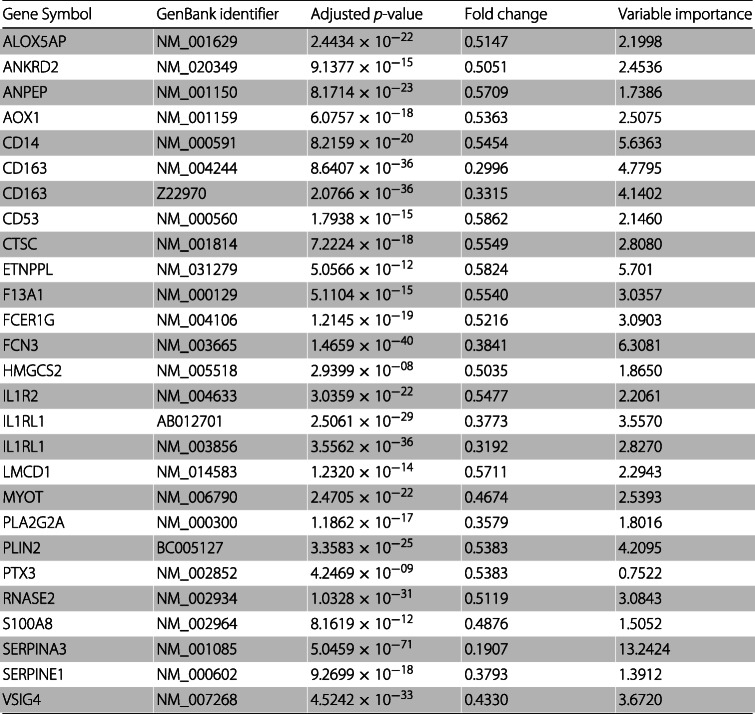
Common down-regulated genes in heart disease obtained when using a fold change cutoff of $\frac {1}{1.6}$

As can be observed in Tables 7 and 8, the values of the adjusted *p*-value are greatly reduced, with 4.5375×10^−05^ being the highest value.

It is worth noticing that the feature set obtained using a fold change cutoff of 2.6 still achieves an accuracy of approximately 93%. This set is composed of 7 GenBank identifiers which correspond to 6 genes. 3 genes are up-regulated (ASPN, SFRP4 and NPPA) and 3 are down-regulated (CD163, IL1RL1 and SERPINA3). We can, also, observe that in Table 7 the mean fold change of gene EIF1AY is higher than 2.6 and that in Table 8 the mean fold changes of genes FCN3 and PLA2G2A are lower than $\frac {1}{2.6}$. However, these genes are not included in the feature set obtained using fold change 2.6 since it is sufficient that a gene is not included in one of the thirty feature sets obtaining using the thirty training sets, to be excluded from the intersection set. We analyzed the thirty feature sets and observed that in four of them gene EIF1AY had a fold change lower than 2.6, in fourteen of them and in three of them genes FCN3 and PLA2G2A, respectively, had a fold change greater than $\frac {1}{2.6}$.

**Fig. 3:**
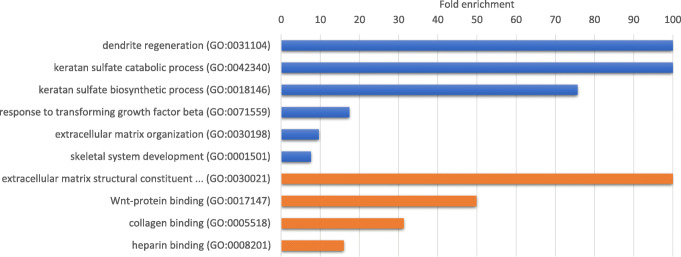
Gene Ontology significant results. Gene Ontology significant results of the up-regulated genes from the gene set with a fold change of 1.6, showing biological process (blue) and molecular function (orange)

To further analyze the feature sets obtained using a fold change cutoff of 1.6, we performed a GO analysis on the up-regulated and down-regulated genes (see Fig. 3 and Additional file 1). Using the PANTHER GO-slim annotation datasets, we observed that the up-regulated genes had the same result, for both the molecular function and the biological process categories, which was related to the Wnt signaling pathway. The down-regulated genes had no statistically significant results. Using the PANTHER complete annotation datasets, we observed an enrichment in up-regulated genes in processes related to tissue regeneration and development and with structural components from the extracellular matrix. The complete results of the GO analysis are presented in Fig. 3 (see Additional file 1 for details on the fold enrichment and FDR values).

**Fig. 4:**
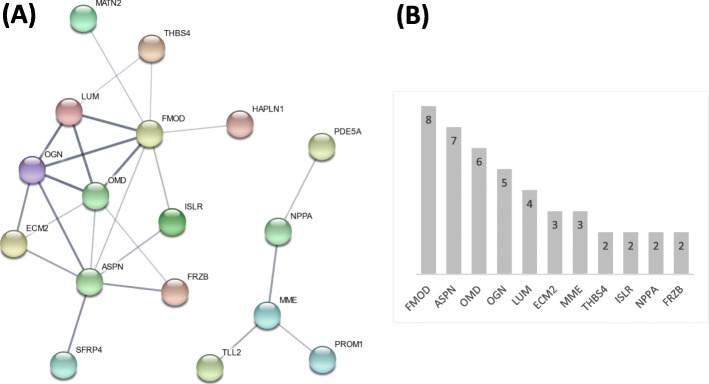
Protein-protein interactions of up-regulated protein coding genes. **a** Protein-protein interactions of the up-regulated protein coding genes from the feature set with fold change cutoff of 1.6, was retrieved from STRING database, resulting in a network of 25 edges between 17 nodes. Each node represents one protein and the edges represent the interactions. The line thickness indicates the strength of data support (text mining, experiments, databases and co-expression were selected from the options), with a default median level of confidence. **b** The number of interactions of the nodes with more than one interaction is represented

To construct the PPI networks, we considered the up-regulated genes from the feature set with a fold change cutoff of 1.6, comprised of 37 genes. The genes DDX3Y, EIF1AY, KDM5D, RPS4Y1, UPS9Y and UTY had no protein match on STRING and were excluded. Out of the 31 proteins found and using only the queried proteins, we retrieved a final network, with 17 nodes (proteins) and 25 edges (interactions), which is shown in Fig. 4. Among the 17, eleven proteins had more than one interaction and 2 were still present in the feature set with a fold change cutoff of 2.6. For the 25 down-regulated genes, we obtained a final network with 15 nodes and 21 interactions (Additional file 2). We found that 10 proteins had more than one interaction.

As a final functional validation step, we used the DisGeNet database to evaluate the disease association status of the genes. We found a total of 7351 associations between 2757 genes and 241 diseases. From the 62 genes of the feature set with a fold change cutoff of 1.6, twenty-four genes were found to be associated with heart related diseases (see Additional file 3). From these 24 genes, five were also in the feature set with a fold change cutoff of 2.6.

## Discussion

In this study, nine microarray datasets were merged in order to identify a GES common to different heart diseases. The term “heart diseases” is often incorrectly used as a synonym of cardiovascular diseases. According to the World Health Organization, as published in the “Global atlas on cardiovascular disease prevention and control” [61], cardiovascular diseases include diseases of the heart, vascular diseases of the brain and diseases of blood vessels. In our study, we focused on heart diseases that affect the structure of the organ (muscle and valves), leaving out all types of vascular diseases, as well as diseases that affect the function or rhythm of the heart (arrhythmias). As depicted in Table 1, the data sets analyzed included different types of cardiomyopathies (diseases of the heart muscle) and diseases of the heart valves, which are often grouped under the term “structural heart diseases”. Genes expressed in atrial and ventricular myocardial tissues from failing and non-failing hearts were analyzed. After applying the methodology described above, we obtained a set of 62 genes with altered expression levels by a fold change cutoff of 1.6, which best discriminates diseased from control samples. To evaluate the performance of the model we used 30 tests sets. Besides this approach, we also evaluated the leave one study out procedure, where the datasets of 8 of the 9 studies are used for selecting the differentially expressed genes and training the model, and the dataset of the remained study is used to evaluate the model’s performance. This process was repeated nine times so that the dataset of each study is once used for independent evaluation. However, the results obtained were over-optimistic, making this procedure unfeasible for our study.

To explore the biological meaning of the results, we performed a functional analysis comprised of GO analysis and PPIs network. Our goal was to understand if the function of the genes obtained was relevant in heart disease and therefore validate our approach to obtain a GES.

The GO analysis of the up-regulated genes revealed an over-representation of several biological processes and molecular functions related to the development of heart tissue and cardiac remodeling after injury (see Fig. 3 and Additional file 1), specifically the involvement of the keratan sulfate metabolic process, which will be discussed later. Interestingly, enrichment in genes that code for proteins involved in the Wnt protein binding and in the regulation of the Wnt signaling pathway was a result highlighted by the PANTHER GO-slim annotation datasets, which are selected by curation. The Wnt-protein is a secreted growth factor involved in signaling. The Wnt signaling pathway, mostly via the beta-catenin pathway, also known as the canonical pathway, has a well-known role in cell proliferation and cell differentiation in tissue development and homeostasis [62]. The role of this pathway in the context of cardiovascular disease has been recently reviewed by Foulquier et. al. [63]. Specifically, the role of screted frizzled-related proteins (SFRPs), which are a family of Wnt modulators, has been studied in this context [64], but remains largely unknown. Considering the down-regulated genes, most of the results are related to the immune system and inflammatory processes. Though the primary event of heart tissue damage is an inflammatory response, the cardiac repair is dependent on the suppression of inflammation to ensure the formation of a scar in the post-infarction response [65–67]. The down-regulation of genes involved in immune and inflammatory response had been observed in a similar study in dilated cardiomyopathy [8].

Next, we obtained a network of PPIs from the STRING database, using the 37 up-regulated genes from the feature set with a fold change cutoff of 1.6 (Fig. 4). We considered that the analysis of the up-regulated genes could be more interesting from the clinical point of view. The analysis of the PPIs network constructed with the down-regulated genes (Additional file 2) showed that the proteins with more interactions were mostly involved in processes related to both the inflammatory response and the immune system (FCER1G, CD14, CD163, and S100A8), which is in agreement with the GO results.

Among the proteins with the highest number of interactions in the up-regulated genes network, we found ASPN and NPPA, which are also found in the smallest feature set with a fold change cutoff of 2.6.

Natriuretic Peptide Precursor A (NPPA) gene encodes the precursor for the hormone atrial natriuretic peptide (ANP). ANP is synthesized and secreted by cardiac muscle cells from the atria in the heart and is a well-established biomarker for cardiovascular disease [68]. According to a recent review [69], it plays a key role in the regulation of cardiovascular volume and pressure homeostasis by inducing natriuresis, diuresis and vasodilation. Over the last four decades, studies have shown that the phenotype associated with NPPA genetic variants and the changes in the circulating levels of ANP reflect its value as a potential therapeutic target for cardiometabolic diseases, including heart failure [70, 71]. Therefore, adding to our study, NPPA gene has been previously identified in GES of cardiac diseases, such as heart failure [72] and dilated cardiomyopathy [8, 73].

Asporin, encoded by ASPN gene, is a glycoprotein from the family of the small leucine rich proteoglycans (SLRP) present in the cartilage tissue. It is a known negative regulator of osteoblast differentiation and might be involved in development of the heart valves [74], being among the structural and extracellular matrix proteins that have putative roles in mitral valve degeneration [75]. Nevertheless, to our knowledge, only a few studies have found an alteration in ASPN gene expression in the context of cardiovascular diseases. In the two studies performed in humans [8, 75], its importance has not been properly discussed. A very recent study performed in mice by Wang et. al.[76] has reported ASPN among the up-regulated genes in a GES of cardiac remodeling. Following those studies, we have identified ASPN as one of the most significant genes altered in heart disease, since it was found among the highest fold change cutoff used and prominent in the PPI network. Together with another group of four proteins from the SLRP family, namely fibromodulin (FMOD), osteomodulin (OMD), osteoglycin (OGN) and lumican (LUM), the importance of extracellular remodeling processes in heart disease is emphasized. These FMOD, OMD, OGN and LUM proteins, also underlined by the PPIs network, are involved in keratan sulfate metabolic, catabolic and biosynthetic processes. Keratan sulfate is a glycosaminoglycan, a structural molecule, mostly found in the extracellular matrix. Keratan sulfate metabolism is involved in the development of heart tissue and has been implicated in heart disease. FMOD and LUM, for example, are increased in heart failure as a response to inflammation and play a role in cardiac remodeling [77, 78]. The findings highlighted by the PPIs network analysis agree with the GO analysis, where the biological processes of the biosynthesis and catabolism of the keratan sulfate was one of the main results.

After the functional study using GO and PPIs network analysis, we searched the DisGeNet database and found that approximately 39% of the genes studied had previously been associated with cardiac-related diseases, including NPPA, SERPINA3, SFRP4, IL1RL1 and CD163, still present in the feature set with a fold change cutoff of 2.6. This final observation strongly validates our results.

## Conclusion

With this study, we were able to successfully identify a GES common to different cardiac diseases, mainly structural heart diseases, and supported our findings by showing their involvement in the pathophysiology of the heart.

According to our findings and given its structural function, we suggest that asporin is likely to be involved in a cardiac tissue mechanism that is up-regulated in response to disease development, rather than having a causal effect. Although this has not yet been demonstrated, it should be further studied.

Regardless of advantages of having a GES, we also consider here that having a small set of markers to distinguish normal from diseased samples can ease their use as a panel for diagnosis or screening. Additionally, such genes can be further investigated in the context of new therapeutic approaches.

Finally, the approach used in this study is suitable for the identification of gene expression signatures and can be extended to different diseases.

## Supplementary information

**Additional file 1** gO results. Gene Ontology significant results of the up-regulated and down-regulated genes from the gene set with a fold change cutoff of 1.6. Only results for *p*-value <0.05 are displayed.

**Additional file 2Figure (B)** The number of interactions of the nodes with more than one interaction is represented.

**Additional file 3** Table with the association status of the 24 genes which were found, using disGeNet, associated with heart related disease.

## Data Availability

The datasets used during the current study are available from the Gene Expression Omnibus repository and their accession numbers are listed in Table 1. The R code used to obtain the results presented in the paper is available at https://github.com/olgafajarda/MergingHD.

## Abbreviations

AUC: Area under the receiver operating characteristic curve
AUCPR: Area under the precision-recall curve
EMBL-EBI: European Bioinformatics Institute
GDA: Gene disease association
GEO: Gene Expression Omnibus
GES: Gene expression signature
GO: Gene ontology
MCC: Matthews correlation coefficient
MDS: Multidimensional scaling
NCBI: national Center for Biotechnology Information
PPI: Protein-protein interactions
PR: Precision-recall
RMA: Robust multichip average
RNA-seq: RNA-Sequencing
ROC: Receiver operating characteristic
VAD: Ventricular assist device

## Footnotes

1 http://pantherdb.org, Version 15.0

2 https://string-db.org, Version 11.0

3 https://www.disgenet.org, Version 6.0
