# Supplementary figures and images for: Merging microarray studies to identify a common gene expression signature to several structural heart diseases
Source: BioData Min. 2020 Jul 8;13:8. doi: 10.1186/s13040-020-00217-8 (PMC7346458; doi:10.1186/s13040-020-00217-8)

**(A)**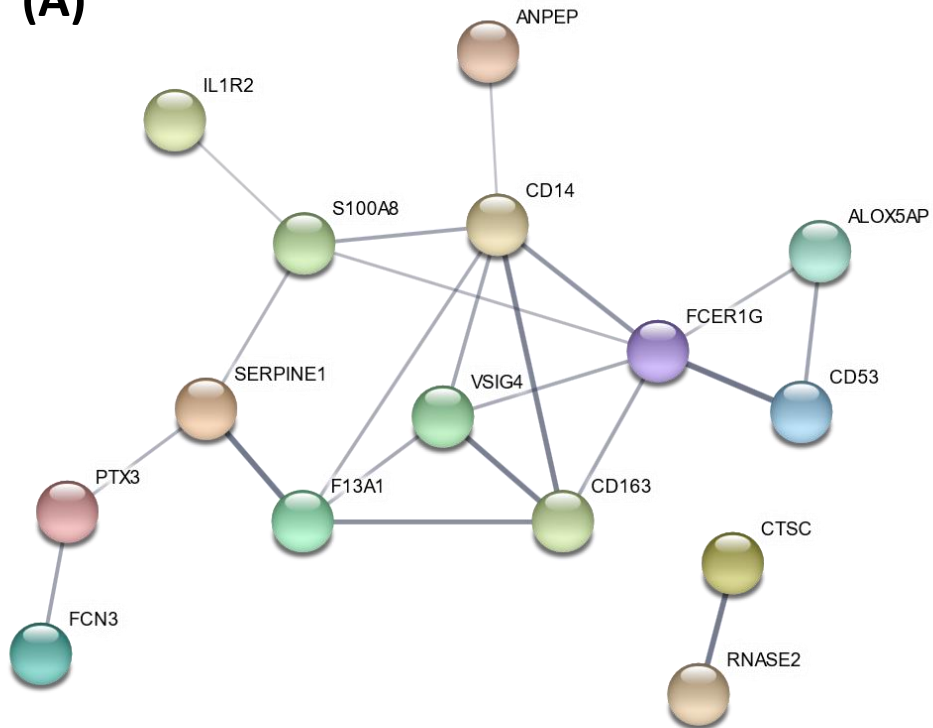**(B)**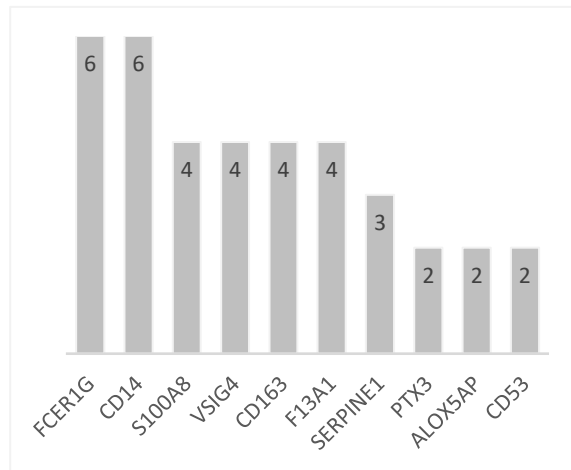

Supplement: Supplementary file 2 — Additional file 2Figure (B) The number of interactions of the nodes with more than one interaction is represented. [file 13040_2020_217_MOESM2_ESM.pdf]
